# Multivessel endovascular therapy for undiagnosed vascular type Ehlers-Danlos syndrome. Successful percutaneous transcatheter coil embolization of hepatic artery pseudoaneurysm with stenting of right renal and iliac arteries in emergency setting

**DOI:** 10.1259/bjrcr.20200025

**Published:** 2020-07-06

**Authors:** Lorenzo Paolo Moramarco, Carlo Alberto Capodaglio, Pietro Quaretti, Nicola Cionfoli, Ilaria Fiorina, Eliana Disabella, Antonio Mauro D'agostino, Mario Urtis, Eloisa Arbustini

**Affiliations:** 1 Department of Radiology, IRCCS Fondazione Policlinico San Matteo, Pavia, Italy; 2 Unit of Interventional Radiology and Department of Radiology, IRCCS Fondazione Policlinico San Matteo, Pavia, Italy; 3 Centre for Inherited Cardiovascular Diseases of Cardio-Thoracic-Vascular Surgery Department, IRCCS Fondazione Policlinico San Matteo, Pavia, Italy

## Abstract

Among Ehlers-Danlos syndromes, the vascular type is the most severe because of its vascular complications. Transcatheter embolization of medium-sized arteries has become the first-line therapy for life-threatening hemorrhage. Ongoing multiple lesions causing hemorrhagic or ischemic complications in the acute phase can challenge patient management. Multivessel endovascular treatment has never been reported. In this study, we report successful single-session treatment by coiling of a ruptured pseudoaneurysm of the hepatic artery with stenting of dissected right renal and iliac arteries in a 46-year-old female. Percutaneous transfemoral approach was gained and sealed with a plug-based closure device. Genetic disease was subsequently confirmed by molecular analysis.

## Introduction

The Ehlers-Danlos syndromes (EDS) are genetically heterogeneous diseases involving the connective tissue, characterized by joint hypermobility, skin hyperextensibility, and tissue fragility. Autosomal dominant vascular EDS (vEDS), previously known as Type IV, is the most feared and lethal form. It is caused by mutations in the COL3A1 gene encoding in the pro-alpha 1 chain of Type III procollagen, widely present in vascular system.^1^ Arterial tears or dissections, rupture of internal organs, pneumothorax, and other clinical criteria characterize the phenotype. Manifestations of vEDS at onset may involve one or more organs.^2^ Conservative management is the mainstay oftherapy.^3,4^ Lethal risks inherent to iatrogenic damage have limited invasive therapeutic options to emergency.^5–7^ Interventionists may face in critical setting several concomitant lesions in undiagnosed vEDS patients.^8^ Endovascular therapy may offer an attractive alternative of multivessel intervention.

## Case presentation

**Figure 1.**
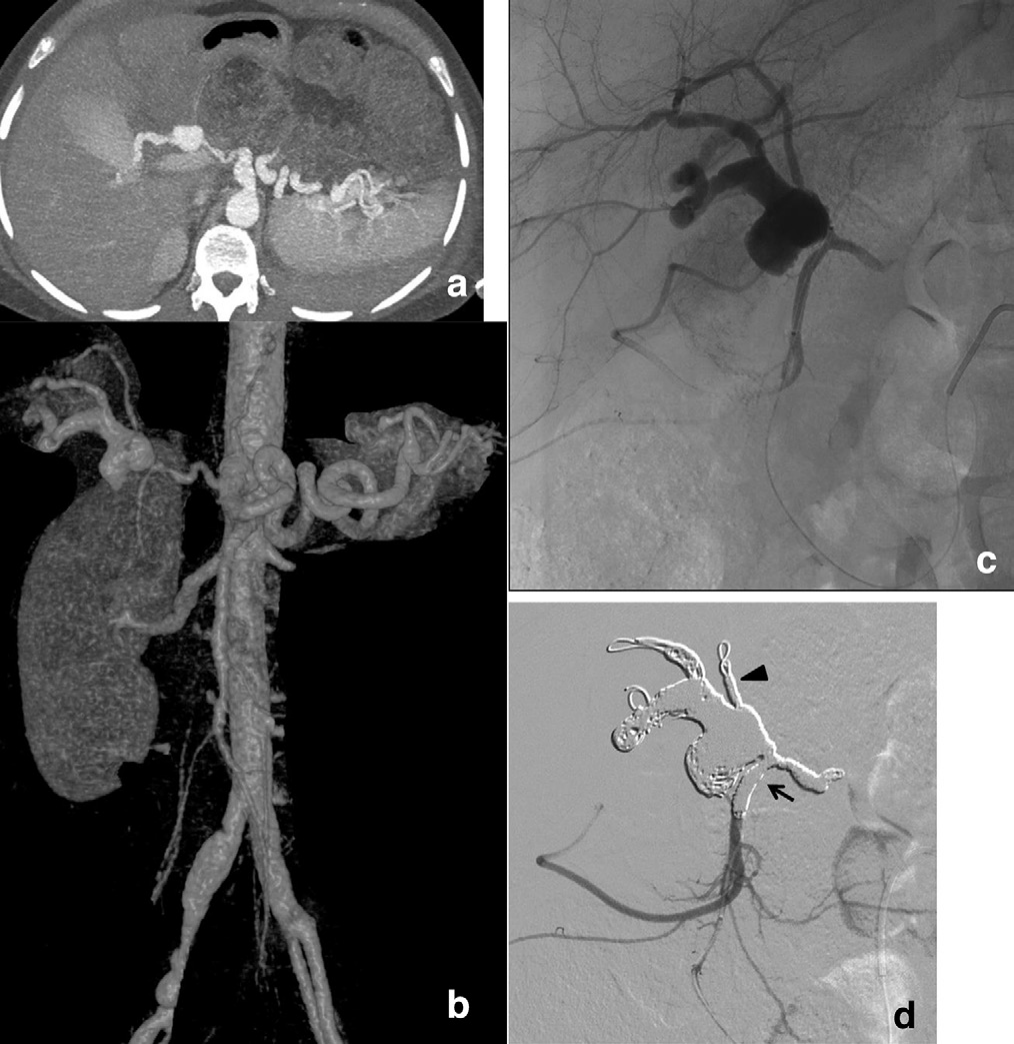
Coronal maximum intensity projection (a) and volume rendered 3D, (b) contrast-enhanced MDCT images showing hepatic artery pseudoaneurysm, the non-navigable celiac trunk, the dissected right renal and iliac axis. (c) Selective retrograde arteriogram via the inferior pancreaticoduodenal arcade vessels: the ruptured pseudoaneurysm involves hepatic artery posterior branch. (d) Completion angiography: complete packing is achieved. Exclusion of the pseudoaneurysm including (arrow) the distal common hepatic artery and the origin of the gastroduodenal artery is shown. Embolization of the anterior branch of the hepatic artery avoids late reperfusion through the backdoor of the pseudoaneurysm (arrowhead).

A 46-year-old female presented with hematuria and left flank pain. Her history showed hypertension, a past hysterectomy, and the sudden death of a brother at 42 years. Multidetector MDCT revealed a left main renal artery dissection causing parenchymal ischemia. Dissections of the celiac trunk, proper hepatic, superior mesenteric, and common iliac arteries were present. 4 days later, she complained of acute dyspnea with hypotension, and tachycardia. Chest X-ray and MDCT showed spontaneous hemothorax and lung collapse on the left side. Additional abdominal findings included worsening of the left renal ischemia and hepatic artery dissection. Dissections of the right renal and splenic arteries with limited polar kidney ischemia and splenic infarction were also observed. A chest drainage was inserted, and 4 units of blood were infused. On the eighth day, the patient showed signs of peritoneal irritation, and hemoglobin level fell to 7 g l^−1^. Repeated MDCT showed the development of a pseudoaneurysm in the common hepatic artery (17 × 17 mm) with hemoperitoneum (Figure 1a and b).

## Treatment

**Figure 2.**
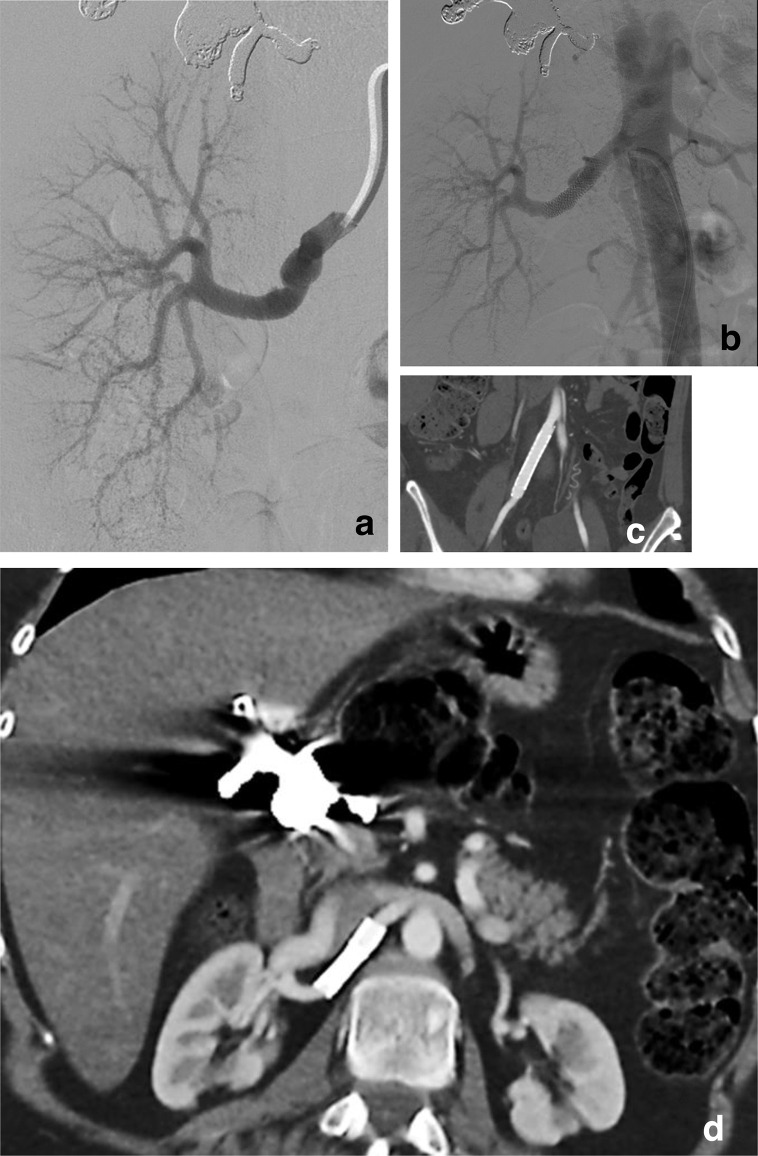
(a) Right renal angiography confirming the dissection. (b) Angiography post-stenting. Carotid Wallstent (Boston Sc., MA) had previously been proven to be compatible with the 6 Fr guiding catheter (c) MDCT at 5 months: patency of the two imbricated self-expandable stents in the right iliac axis. (d) Patent renal stent and consistency of the hepatic artery cast without reperfusion.

Emergent angiography was performed on Philips Allura FD20. A 6 Fr valved sheath (Pinnacle,Terumo,Japan) was inserted in the right common femoral artery. Catheterization of the celiac trunk with a 5 Fr pre-curved catheter (Glidecath, Terumo, Japan) confirmed a tiny and angulated residual lumen of the proper hepatic artery feeding a ruptured pseudoaneurysm (20 x 14 mm). Attempts to pass beyond the lesion with 0.014 microguidewires failed. The superior mesenteric artery had to be engaged. The duodenal pancreatic arcade was navigated through a retrograde approach with a high-flow 0.027” microcatheter (Lantern, Penumbra, CA) (Figure 1c). The release of more than 600 cm of soft platinum Ruby coils (12 coils) (Penumbra, CA) achieved the complete packing of the aneurysm sack with occlusion of front and back doors (Figure 1d). The right renal artery was then opacified (Figure 2a). Despite repeatedly entangling in the false lumen, a 0.014 guidewire was finally inserted into the true lumen under road mapping. After digital calibration of the artery, two overlapping self-expandable stents, 7 x 30 mm Carotid Wallstent (Boston Sc., MA), were deployed through a 6 Fr guiding catheter Mach I (Boston Sc.,MA) (Figure 2b). Post-dilation was unnecessary. As right renal stenting was deemed sufficient to preserve global kidney function, the dissection of the contralateral artery was left untreated because of the technical challenges and unfavorable risk/benefit ratio. Before the removal of the 6 Fr sheath, two 10 × 60 mm nitinol stents (Smartflex, Cordis) were deployed to repair the dissection in the right iliac axis. Finally, groin hemostasis was achieved with Femoseal (Terumo, Japan). Procedure duration and fluoroscopy time were 102 and 38 min respectively. Cumulative kerma–area-product (P_KA_) was 4.8 Gy∙cm2 (1.53 Gy∙cm2 for stenting).

## Outcome and follow-up

The patient was transferred to ICU. After multidisciplinary team consultation, a low dose mono antiplatelet regimen was prescribed due to the renal stenting and the other untreated dissected arteries. 3 weeks later, she was discharged on therapy with beta-blocker and ASA 100 mg daily. Due to suspicion of inherited connective tissue disease, genetic counseling was suggested. The novel COL3A1 variant c.2824–3*T* > G was identified by means of Next Generation Sequencing analysis in the proband and in the mother, while the four unaffected living family members tested negative. A surveillance program was scheduled. CT scan, performed 5-months later for relapsing pneumothorax, conservatively managed, showed consistency of the hepatic coiling without signs of reperfusion, regular patency of the stented arteries, right kidney hypertrophy, and inferior cortical scars on the left kidney (Figure 2c and d). Biochemical values measured within normal range. At 13 months follow-up, the patient remained asymptomatic.

## Discussion

Arterial dissection and rupture is the primary cause of morbidity and mortality in vEDS patients. Vascular lesions can precede clinical events leading to sudden death at a young age.^9^ Diagnosis is based upon clinical criteria, confirmed by genetic testing.^1^ At onset, patients can be aware of their vEDS in only 4–26% of cases, and family history may be positive in only 31%.^10^ Genotype-phenotype correlation is often elusive, and typical vEDS signs as hyperelasticity and hypermobility can be absent at clinical examination.^10^ Management of the patient in the acute phase is always challenging. Concurrent multiple arterial lesions can be detected by contrast enhanced cross-sectional-imaging. The favored therapeutic option for life-threatening hemorrhage due to rupture of medium-size artery is transcatheter embolization.^4,10,11^ Clinical course remains unpredictable, and secondary accidents in distant vascular territories may be impending and fatal.^7^ A protocol of “permissive hypotension” has been suggested in which hypotension is permitted in the acute phase as well as avoidance of inotropic agents and judicious use of i.v. fluids.^3^ In attempt to define the period of high risk for a new accident after index event, a 2 weeks time span has been estimated on the basis of neurologic experience.^8^ For vEDS patients, whether to intervene solely in emergency or even in elective circumstances has been a disputed issue. Abstention from treatment is recommended for asymptomatic patients, as the rate of angiography-related complications (up to 67%) is well-known.^5^ This attitude, however, might not guarantee a positive clinical evolution. Oderich et al reported about untreated asymptomatic lesions in six patients. All experienced adverse outcome, including two deaths.^4^ Conversely, the literature regarding the safety of endovascular therapy in vEDS is expanding.^11^ Lum et al reported 48 procedures in 26 EDS patients.^11^ Overall, three coil-embolizations, including one hepatic pseudoaneurysm, were successfully undertaken in five proven vEDS patients.^11^ Recently, Okada et al. carried out seven emergent transcatheter coiling and/or glue embolizations in five consecutive vEDS patients.^12^ All have succeeded with one minor access complication.^12^ Several factors have been advocated for this achievement.^11,12^ In addition to the improvement in quality of the devices that are being used, the impact of cross-sectional imaging on clinical decision-making has been mentioned.^6^ In the present case, following unilateral renal artery dissection, a known clinical onset of vEDS, a ruptured hepatic pseudoaneurysm was clearly depicted by MDCT.^13,14^ A guiding catheter was preferred to long sheath for superior steerability and trackability. A cardiologic stent-graft deployment was firstly conceived, but celiac trunk dissection and sharp angulation of proximal hepatic artery halted forward advance of guidewire. Retrograde embolization was then optioned. Glue and DMSO-based liquid agents were ruled out due to the risk of non- target embolization. Ultimately, choice fell on Ruby coils. They are extremely soft, high-volume packing, platinum coils derived from technology developed for neurovascular interventions, and length up to 60 cm is provided. Owing to their softness, high packing density of sac aneurysm was gained without vessel perforation. The detachment of coils was controlled by a dedicated device, thus allowing to check coil-deployment before release. In the same session, the right renal and iliac artery were also stented. The clinical decision to embolize the culprit hemorrhagic lesion was undisputable, and its technical execution was straightforward and expeditious. Stenting was judged to add little risk and radiation dose to procedure. (Supplementary Materials 1 and 2) Both stentings were accomplished without post-dilation or exchange of the introducer, thus minimizing the risk of femoral damage.^13^ The radiation dose did not exceed the reference P_KA _threshold of 5 Gy /cm2.^15^ Hemostasis was achieved with a plug-based occlusion device. In vEDS, effective femoral closure with Angiosealhas been described after carotid stenting.^16,17^

Supplementary Material 1.

Supplementary Material 2.

## Conclusion

We reported successful transcatheter embolization with coiling of a ruptured hepatic artery pseudoaneurysm followed in the same session by stenting for dissection of the right renal and iliac arteries in a patient subsequently diagnosed as vEDS. No manipulation of the sheath was necessary and hemostasis was achieved with a plug-based device.

## Learning points

1. Transcatheter embolization is the first-line treatment of hemorrhagic lesions of medium-sized artery in vEDS patients.
2. A percutaneous approach can be cautiously performed in vEDS patients. Minimal manipulation of the vascular access once in place and careful use of the plug-assisted device for hemostasis may minimize the risks of complications at the access site.
3. Findings of multivessel lesions in the acute phase of vEDS are not uncommon. Genetic counseling is warranted if vEDS is still undiagnosed and a heritable connective disease is suspected.
