# Supplementary figures and images for: Multivessel endovascular therapy for undiagnosed vascular type Ehlers-Danlos syndrome. Successful percutaneous transcatheter coil embolization of hepatic artery pseudoaneurysm with stenting of right renal and iliac arteries in emergency setting
Source: BJR Case Rep. 2020 Jul 6;6(4):20200025. doi: 10.1259/bjrcr.20200025 (PMC7709055; doi:10.1259/bjrcr.20200025)

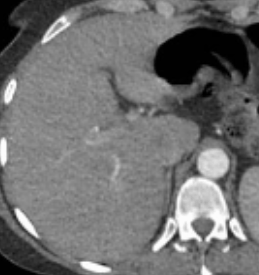

Supplement: Supplementary Material 1. [file bjrcr.20200025.suppl-01.gif]

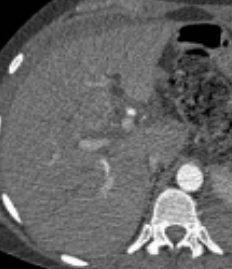

Supplement: Supplementary Material 2. [file bjrcr.20200025.suppl-02.gif]
